# Kinlessness and Social Isolation at the End of Life: Cross-National Comparisons

**DOI:** 10.1093/geroni/igaa057.2035

**Published:** 2020-12-16

**Authors:** Christine Mair, Katherine Ornstein, Feinian Chen

## Abstract

Nearly all older adults cross-nationally rely on close family members to facilitate care at the end-of-life, but recent demographic shifts toward declining fertility and marriage rates have yielded an unprecedented increase in older adults who lack traditional family ties (“kinless”). “Kinless” older adults may be at risk for social isolation, lack of caregiving options, and poorer end-of-life outcomes, and these risks may be buffered or exacerbated by social and economic resources as well as country context. Because the potential impact of “kinlessness” on end-of-life outcomes cross-nationally is unknown and virtually unstudied, this symposium composed of overlapping, interdisciplinary research teams investigate if and how “kinlessness” is associated with end-of-life outcomes cross-nationally. Mudrazija and Ornstein analyze a sample of older European adults (SHARE) to predict location of death (e.g., home deaths) by family size, living alone, and size of social network. Plick, Ankuda, and Ornstein examine a sample of older American decedents (HRS) to predict end-of-life outcomes (e.g., hours of care received, location of death). Mair, Ornstein, Aldridge, and Thygesen explore associations between family structure and multiple end-of-life outcomes (e.g., hospitalizations, ICU visits, medical treatment) among the population of decedents aged 50+ from Denmark. Finally, Mair, Ornstein, Calvo, Donoso, and Medina explore family structure, social isolation, loneliness, and end-of-life outcomes among a sample of older adults from 20 countries (Gateway to Global Aging). These papers will be discussed by Feinian Chen, a family sociologist and social demographer who specializes in cross-national comparisons of older adults’ family structures, country context, and health.

